# Charcot on the the Relief of Hysterical Seizures by Compression of the Ovaries

**Published:** 1875-09

**Authors:** 


CHARCOT ON THE RELIEF OF HYSTERICAL SEIZURES BY COMPRESSION OF THE OVARIES.—According to Charcot most hysterical seizures are preceded by an aura starting from one or both of the ovaries, and he finds that pressure on the organ indicated causes immediate arrest of the seizure. He illustrated this action upon a patient in the Saltpetriere affected with hystero-epilepsy. The seizure recurred, however, the moment the pressure was taken off. In order to keep up the pressure for a longer time than would be possible with the unaided hands, he recommends an apparatus like a tourniquet. The pressure is to be made in the situation and direction requisite in compressing the iliac artery, and, in fact, this artery will be felt pulsating under the finger. *Gaz. Med. de Paris.*

